# A Linear Surface Epitope in a Proline-Rich Region of ORF3 Product of Genotype 1 Hepatitis E Virus

**DOI:** 10.3390/v8080227

**Published:** 2016-08-18

**Authors:** Yonglin Yang, Shaoli Lin, Yuchen Nan, Zexu Ma, Liping Yang, Yanjin Zhang

**Affiliations:** 1 Molecular Virology Laboratory, VA-MD College of Veterinary Medicine and Maryland Pathogen Research Institute, University of Maryland, College Park, MD 20742, USA; easing@163.com (Y.Y.); lsl1990@umd.edu (S.L.); nanyuchen2015@nwsuaf.edu.cn (Y.N.); zexuma@umd.edu (Z.M.); ylp521@umd.edu (L.Y.); 2 Nanjing Red Cross Blood Center, Nanjing 210003, Jiangsu, China; 3 College of Veterinary Medicine, Northwest A&F University, Yangling, Xi’an 712100, Shaanxi, China

**Keywords:** hepatitis E virus, HEV, ORF3, VP13, PXXP motif, linear epitope, monoclonal antibody

## Abstract

Hepatitis E virus (HEV) is one of the viral pathogens causing hepatitis in humans. HEV open reading frame 3 (ORF3) encodes a small multifunctional protein (VP13), which is essential for HEV infection. In this study, a linear epitope was identified in a polyproline (PXXP) motif from VP13 of genotype 1 HEV by using a monoclonal antibody. The epitope was detected in enzyme-linked immunosorbent assay (ELISA), immunoblotting and immunofluorescence assays. Epitope mapping showed that the epitope locates in a proline-rich region containing a PXXP motif in amino acid residues 66-75 of VP13. The epitope was also detected in HEV-infected liver cells and reacted with genotype 1-specific antibodies in an HEV-positive human serum sample. The results demonstrated that the epitope in the PXXP motif of the genotype 1 VP13 is linear and surface-oriented, which should facilitate in-depth studies on the viral protein and HEV biology.

## 1. Introduction

Hepatitis E virus (HEV) is a single-stranded positive-sense RNA virus in the family of *Hepeviridae* [1]. HEV is one of the viral pathogens mainly causing acute hepatitis in humans, seen in both sporadic cases and epidemic outbreaks in various tropical and subtropical regions of the world. Sporadic HEV infections also occur in non-endemic regions [2,3]. One unique feature of HEV infection is that it can lead to fulminant hepatitis with a case fatality rate of up to 30% in pregnant women [4,5]. Recently, chronic and persistent HEV infections in immunocompromised individuals, including organ transplant recipients and patients with leukemia, lymphoma and human immunodeficiency virus infection and acquired immune deficiency syndrome (HIV/AIDS), have been reported in industrialized countries [4].

The HEV genome is approximately 7.2 kb in length and encodes three open reading frames (ORFs) [6]. ORF1 is the largest ORF and encodes a polyprotein, which is possibly cleaved into the putative nonstructural proteins involved in HEV replication. ORF2 is the second largest ORF and encodes the capsid protein, the major structural protein in the HEV virion. ORF3 is the smallest ORF and encodes a multi-functional phosphoprotein VP13 with a molecular mass of approximately 13 kDa, which was found to be essential for HEV infection in macaques and pigs [7,8]. A single bicistronic RNA was found to encode both ORF2 and ORF3, whose initiation codons are closely spaced in two different reading frames [9]. 

HEV strains are highly diverse in sequence and those strains infecting humans are classified into four genotypes belonging to the genus *Orthohepevirus* [10,11]. HEV strains of genotypes 1 and 2 are restricted to humans with no known animal reservoir, whereas HEV strains of genotypes 3 and 4 are zoonotic and infect several animal species in addition to humans [12,13].

A number of studies showed that VP13 plays roles in cellular signaling pathways [13], interacts with microtubules [14] and assists virion release [15,16,17]. The VP13 enhances the retinoic acid-inducible gene 1 (RIG-I) signaling via extending its half-life and increasing its ubiquitination during RIG-I activation [18]. The VP13 enhancement of RIG-I signaling appears to be genotype-specific; particularly, VP13 from genotype 1 and 3, but not VP13 from the other two genotypes, can do so. These data indicate that VP13 may play important roles in HEV virus-cell interactions and further study of VP13 in HEV biology and pathogenesis is needed.

In this study, we identified a linear surface-oriented epitope on VP13 of genotype 1 HEV using a monoclonal antibody (Mab). The epitope was located in a proline-rich region of VP13, which contains a PXXP motif, a structure known to bind to SRC homology 3 (SH3) domains [19,20]. This finding of a genotype-specific linear and surface epitope of VP13 should facilitate further studies of the viral protein and HEV biology. 

## 2. Materials and Methods

### 2.1. Cells and Transfection

HEK293 cell line was obtained from ATCC (Manassas, VA, USA, CRL-1573). S10-3 cell line was provided by Suzanne Emerson at the National Institutes of Health (Bethesda, MD, USA) [21]. Both cell lines were maintained in Dulbecco’s Modified Eagle’s Medium (DMEM) supplemented with 10% fetal bovine serum (FBS). The cells were transfected with VP13 plasmid by using FuGENE^®^ HD Transfection Reagent (Promega, Madison, WI, USA). At 48 h after transfection, the cells were collected for VP13 protein detection.

Full-length RNA of HEV Sar55 was obtained by in vitro transcription from replicon plasmid pSK-E2 as described previously [18,22]. Transfection of S10-3 cells with HEV RNA was performed using DMRIE-C reagent (Invitrogen, Grand Island, NY, USA).

### 2.2. Plasmids

Cloning HEV ORF3 of genotype 1 Sar55 strain to vector pCAGEN and PVL847 with the maltose-binding protein (MBP) tag was previously reported [22,23]. Construction of ORF3 plasmids of genotype 2, 3 and 4 was described previously [18]. Deletion and point mutation constructs of ORF3 were cloned into pVenus-C1 vector as described [14,18]. Cloning of VP13-D1 to D4 domains was described previously [14]. For cloning of VP13-D5 to D10 of genotype 1 VP13 into pVenus-C1 vector, PCR amplification was done with respective primers (Table 1) and ligated into the vector at EcoRI and XhoI sites. For cloning the fragments encoding amino acid (aa)66–75 of the VP13 of the four genotypes, annealing of two complementary oligos (Table 1) was done before ligated into the pVenus-C1 vector at EcoRI and XhoI sites. After construction, the sequences of final plasmids were confirmed by DNA sequencing.

### 2.3. Expression and Purification of VP13

Purification of VP13 protein from bacterial expression system was performed as previously described [23]. Briefly, Luria-Bertani (LB) broth was used to culture BL21 bacteria transformed with PVL847-MBP-VP13 plasmid, which encodes MBP-VP13 fusion protein. The bacteria were induced with 0.2 mM isopropyl β-d-1-thiogalactopyranoside (IPTG) at 28 °C and harvested 2 h later. The cells were pelleted and lysed by ultrasound sonication on ice. The MBP-VP13 fusion protein in the supernatant sample was purified with MBPTrap HP columns (GE Healthcare Life Sciences, Pittsburgh, PA, USA) following the manufacturer’s instructions. The purified MBP-VP13 was subjected to sodium dodecyl sulfate-polyacrylamide gel electrophoresis (SDS-PAGE) and SYPRO Ruby protein gel straining (Bio-Rad, Hercules, CA, USA), and confirmed by Western blotting (WB). The purified VP13 was used to coat 96-well enzyme immunoassay (EIA) plate at 2 μg·mL^−1^ to screen for hybridoma clones that secrete VP13 antibodies.

### 2.4. Immunization and Monoclonal Antibody Preparation

Specific pathogen free female BALB/c mice at age of 6–8 weeks were bought from Yangzhou University (Jiangsu, China) and maintained in the Animal Care Center of Nanjing Medical University (Jiangsu, China). All animal studies were performed according to protocols approved by the Animal Welfare Committee of Nanjing Medical University. The mice were injected with VP13 plasmid (pCAGEN-VP13) DNA solution through tail vein. The solution was a mixture of 10 μg plasmid DNA and TransIT^®^—EE Delivery Solution (Mirus, Madison, WI, USA) incubated at room temperature for 20 min and diluted with 0.9% NaCl to a final volume of 2 mL. The mice were injected with the VP13 plasmid three times at two week intervals and monitored for antibody production. A final boost was given with 200 μg of purified VP13 protein by intraperitoneal injection one week before the mice were sacrificed for spleen cell isolation and fusion with SP2/0 mouse myeloma cells according to standard procedure described previously [24]. Hybridomas were selected with hypoxanthine-aminopterin-thymidine (HAT) medium for one week and maintained with hypoxanthine-thymidine (HT) medium thereafter. Supernatant samples of the hybridomas were tested by enzyme-linked immunosorbent assay (ELISA). Sixteen positive clones were identified and selected for three rounds of subcloning. The hybridoma clones that were positive in ELISA in all wells during the final round subcloning were selected for further analysis. Finally, one hybridoma clone 3C3 against VP13 was established and cultured to produce Mab for further analysis.

### 2.5. Enzyme-Linked Immunosorbent Assay (ELISA)

The purified MBP-VP13 protein was used to coat EIA plate (Corning, New York, NY, USA) at a concentration of 2 μg·mL^−1^ diluted in phosphate-buffered saline (PBS) pH 7.2. The plate was then blocked with 3% bovine serum albumin (BSA) in PBS. Purified MBP-X protein (the macro domain of HEV ORF1 product) was included as a negative control. The VP13 Mab was added to the plate. Goat anti-mouse IgG-horseradish peroxidase conjugate (Rockland Immunochemicals Inc., Limerick, PA, USA) was used to detect the binding VP13 antibody on the plate. Specific reaction was revealed with tetramethylbenzidine (TMB) substrate (Thermo Fisher Scientific, Waltham, MA, USA) and absorbance at 450 nm was detected. 

To test the reactivity of VP13 peptides from four HEV genotypes with VP13 Mab and human serum samples (provided by Dr. J. Meng in Southeast University, Nanjing, China), the respective peptides were used to coat EIA plate at 10 μg·mL^−1^ diluted in PBS. The VP13 antibody or human serum dilutions were added to the plate, followed by addition of goat anti-mouse or human IgG-horseradish peroxidase conjugate (Rockland Immunochemicals Inc.) and TMB substrate as described above.

### 2.6. Western Blotting

Protein separation by SDS-PAGE, and detection by WB were conducted as described previously [25]. Antibodies used in the WB were rabbit anti-MBP antibody (Rockland Immunochemicals Inc.), mouse anti-YFP (yellow fluorescent protein) monoclonal antibody (Rockland Immunochemicals Inc.), mouse anti-tubulin monoclonal antibody (Sigma-Aldrich Corp., St. Louis, MO, USA), homemade monoclonal anti-VP13 clone 3C3 (this study) and goat anti-mouse IgG or goat anti-rabbit IgG conjugated with horseradish peroxidase (Rockland Immunochemicals Inc.).

### 2.7. Immunofluorescence Assay (IFA)

IFA was carried out as previously reported [18]. The homemade monoclonal anti-VP13 clone 3C3 and goat anti-mouse IgG Dylight 488 or Dylight 549-conjugated (Rockland Immunochemicals Inc.) were used in IFA. SlowFade Gold antifade reagent containing 4′6′-diamidino-2-phenylinodole (DAPI) (Invitrogen) was used for slide mounting before observed under fluorescence microscopy.

### 2.8. Statistical Analysis

Differences between treatment samples were assessed by Student’s *t*-test. A two-tailed *p*-value of less than 0.05 was considered significant.

## 3. Results

### 3.1. A VP13 Mab only Reacts with VP13 of Genotype 1 HEV

**Figure 1:**
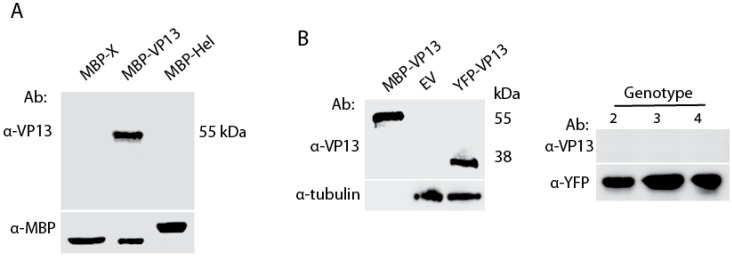
Genotype 1 VP13 protein reacts with the monoclonal antibody (Mab) in western blotting (WB). (**A**) Detection of purified maltose-binding protein (MBP)-VP13, but not MBP-X or MBP-Helicase. MBP detection was done as a control; (**B**) Detection of only genotype 1 VP13 in lysate of HEK293 cells transiently transfected with YFP-VP13 plasmids or empty vector (EV). Purified MBP-VP13 was included as a positive control. Ab: antibody.

**Figure 2:**
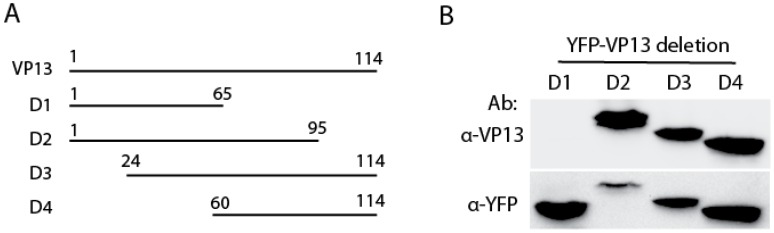
The region of amino acid 66–95 of VP13 specifically reacts with the Mab. (**A**) Illustration of VP13 and truncation mutants. D1, D2, D3 and D4 indicate VP13 deletion constructs. The numbers above lines indicate positions of amino acids in VP13; (**B**) WB of VP13 truncation mutants with the Mab. HEK293 cells were transfected with YFP-VP13 plasmids of the four VP13 truncation fragments. Ab: antibody.

Monoclonal antibodies were generated against VP13 of the genotype 1 HEV strain Sar55 [26] as previously described [24] with modifications described in Methods. A hybridoma clone 3C3 was obtained and characterized. Result showed that the VP13 Mab reacted with MBP-VP13 of Sar55 in immunoblotting (Figure 1A), whereas it did not react with MBP-X or MBP-helicase proteins of HEV that were similarly purified from a bacterial expression system. Next we tested whether the Mab reacted with VP13 from mammalian cell lysate samples. The YFP-VP13 expressed in HEK293 cells was detected by this Mab (Figure 2B). To determine whether the VP13 Mab reacts with VP13 protein of other three HEV genotypes that infect humans, we transfected HEK293 cells with YFP-VP13 plasmids of genotype 2 to 4 as described [18]. WB results showed that the Mab failed to react with VP13 of the type 2, 3, and 4, whereas a YFP antibody detected all of them (Figure 2B), which suggests that the epitope targeted by the Mab is not conserved in VP13 of these three HEV genotypes. As the VP13 protein in immunoblotting was detected under denaturing conditions, the results indicated that the Mab reacts with a linear epitope in VP13 of genotype 1 HEV.

### 3.2. The Linear Epitope Spans Residues aa66-75 of VP13 Protein

To define the VP13 domain that the Mab interacts with, we transfected HEK293 cells with four truncated VP13 plasmids as described previously [14]. WB result showed that VP13-D2 to D4 truncation fragments reacted with the Mab, but not VP13-D1 (Figure 2A,B), which suggests that the VP13 domain interacting with the Mab is located in aa residues 66–95 as VP13-D1 truncation construct spans aa1–65 and VP13-D2 spans aa1–95. Both VP13-D3 and VP13-D4 contain the residues aa66–95.

Analysis of VP13 using program Protean (DNAStar Inc., Madison, WI, USA) suggests that residues aa83–91 are hydrophilic and potentially surface-oriented. We cloned the peptide aa83–91 into pVenus-C1 vector and over-expressed the fusion protein. WB did not detect any specific interaction between the Mab and the fusion protein (result not shown), which suggests that peptide aa83–91 is not the linear epitope targeted by the Mab.

**Figure 3:**
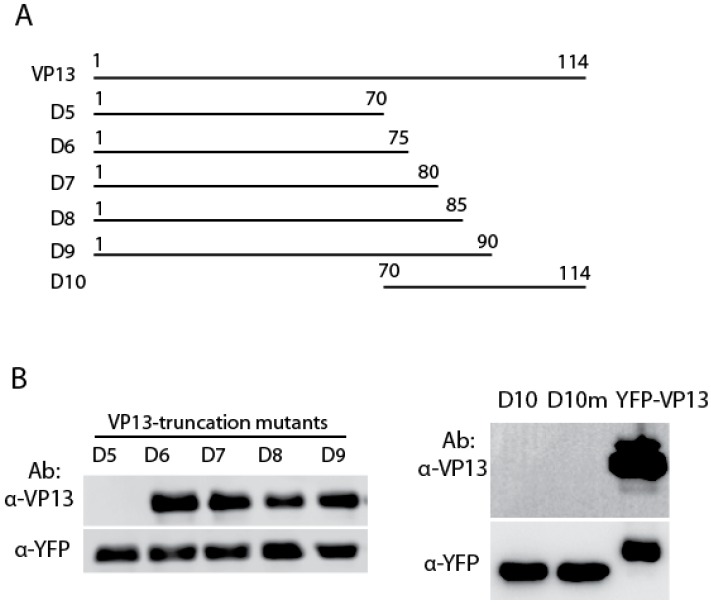
Epitope locates in residues aa66-75 of VP13. (**A**) Illustration of VP13 and truncation mutants. D5 to D10 indicate VP13 deletion constructs. The numbers above lines indicate positions of amino acids in VP13; (**B**) Detection of truncation mutants D6 to D9 but not D5 and D10. HEK293 cells were transfected with the VP13 truncation constructs and harvested for immunoblotting with VP13 Mab at 1:5000. D10: aa71S (type 1); D10m: aa71L (type 2). Ab: antibody.

Six VP13 truncation plasmids were constructed to identify the linear epitope on aa66–95 that interacts with the VP13 Mab (Figure 3A). WB showed that VP13-D6 to D9 truncation fragments reacted with the Mab, but VP13-D5 and D10 did not (Figure 3B), which suggests that the epitope is located between aa66–75 as VP13-D5 spans aa1–70 and VP13–D6 spans aa1–75. A mutant VP13-D10 with a mutation of residue Ser70 to Leu70 was also constructed, but did not react with the Mab (Figure 3B).

### 3.3. The Residue Methionine 70 Is Essential for the Interaction of the Linear Epitope with the Mab

**Figure 4:**
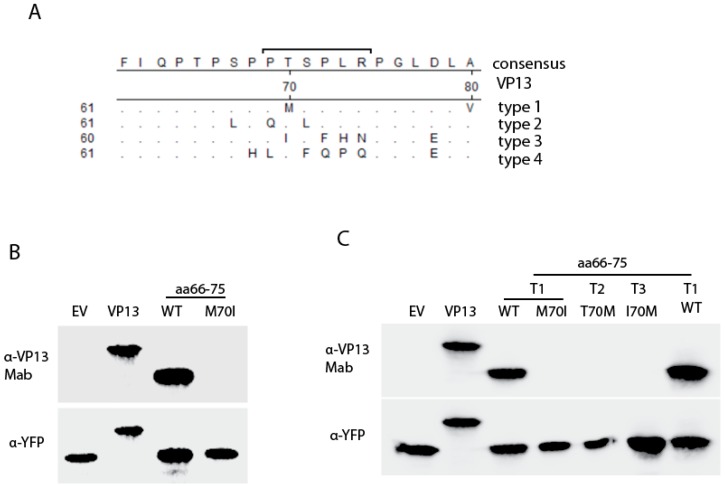
The M70 residue of the epitope is essential for the VP13 Mab binding. (**A**) Alignment of amino acid sequence of VP13 from the four genotypes (genotype 1 Sar55 strain (GenBank accession #AF444002), genotype 2 Mexican strain (GenBank accession# M74506), genotype 3 Kernow-C1 strain (GenBank accession# HQ709170), and genotype 4 JAK-Sai strain (GenBank accession# AB074915). The alignment of residues 61 to 80 is shown. The numbers between the two lines indicate amino acid number of VP13. Identical residues to consensus sequence are shown as “.” and different residues are shown. The PXXP motif is indicated by a solid line above the residues 69–74; (**B**) The VP13 Mab binds to aa66–75 peptide and M70 residue is essential for interaction. HEK293 cells were transfected with the VP13, VP13aa66–75 and VP13aa66–75-M70I, and harvested for immunoblotting with VP13 Mab; (**C**) Mutation of residue 70 of type 2 and 3 VP13 to methionine is unable to have the peptide aa66–75 of type 2 and 3 interact with the VP13 Mab. WT: wild type.

The results above indicate that the linear epitope is located on residues aa66–75 of VP13. Alignment of VP13 of the four HEV genotypes that infect humans showed that this epitope overlaps with a proline-rich region (Figure 4A). The proline-rich region contains a PXXP motif, a structure known to interact with SH3 domains [19,20]. The sequence alignment showed that residue 70 is methionine in type 1 VP13, threonine in type 2 and 4 VP13, and isoleucine in type 3 VP13. To confirm the linear epitope for the Mab binding, we cloned the peptide aa66–75 and expressed it as a YFP fusion protein. A mutant peptide, with methionine 70 changed to isoleucine (M70I), was also cloned as residues 66–71 between type 1 and 3 VP13 are the same except for residue 70. WB result showed that the peptide aa66–75 reacted with the Mab, but not the mutant M70I (Figure 4B). The result indicates that M70 is an essential amino acid for the Mab interaction with the peptide.

To confirm this, we constructed peptide aa66–75 mutants of type 2 and 3 at residue 70, resulting T70M for type 2 and I70M for type 3. Both were expressed as YFP fusion protein. The peptide aa66–75 of type 4 VP13 was not used for mutant construction as seven of the ten residues are different from type 1. WB result showed that only the wild type peptide 66–75 of type 1 VP13 interacted with the Mab, but mutants of type 2 (T70M) and 3 (I70M) VP13 did not (Figure 4C).

### 3.4. IFA Detection of the Linear Epitope in HEV-Infected Cells Using the Mab

**Figure 5:**
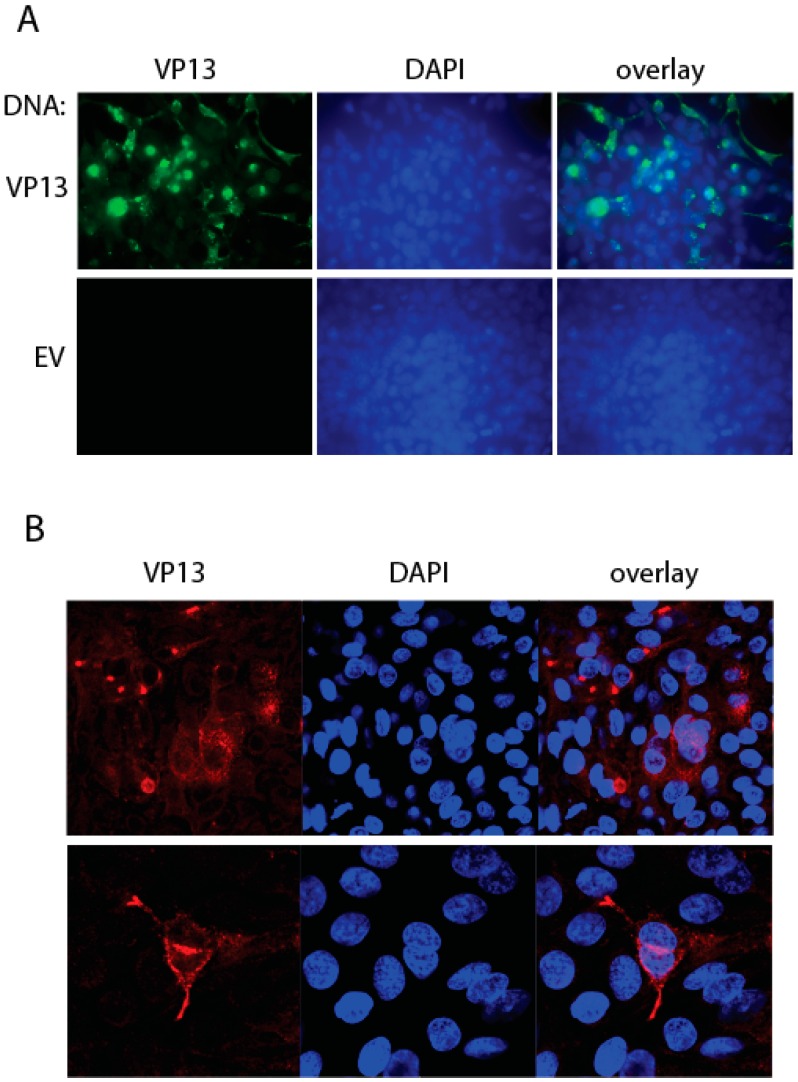
Detection of the linear epitope in HEV-infected cells by immunofluorescence assay (IFA). (**A**) IFA of HEK293 cells transfected with VP13 plasmid or empty vector (EV). Green fluorescence on the left panel indicates VP13 protein. Nuclear DNA shown in the middle panel was counterstained with 4′6′-diamidino-2-phenylinodole (DAPI, blue fluorescence). An overlay is shown on the right; (**B**) Detection of the VP13 epitope in HEV-infected S10-3 cells. The cells were transfected with HEV Sar55 RNA. Red fluorescence on the left panel indicates VP13 protein. Nuclear DNA shown in the middle panel was counterstained with DAPI (blue fluorescence).

The results above showed that a linear epitope in VP13 was detected by the Mab. To test if it can be detected by IFA, we transfected HEK293 cells with VP13 plasmid and conducted IFA with the Mab. The results showed that the VP13 protein in the transiently transfected cells was detected by the Mab, whereas no specific fluorescence was observed in cells transfected with empty vector (Figure 5A), which suggests that the linear epitope is oriented on the protein surface and accessible by the Mab.

To determine if the VP13 epitope is detectable in HEV-infected liver cells, we transfected S10-3 cells with HEV RNA from Sar55 replicon. IFA with the Mab showed that the VP13 epitope was detected in the HEV-infected cells (Figure 5B). The pattern was consistent with previous observation using a rabbit polyclonal antibody against VP13 [18].

### 3.5. The Peptide aa66–75 Is Genotype-Specific in Reacting with HEV-Positive Human Serum Samples

**Figure 6:**
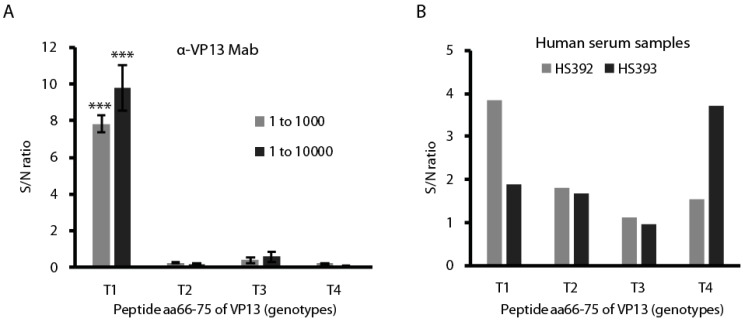
The VP13 aa66–75 peptide reacts with genotype-specific antibodies in human serum samples in ELISA. (**A**) Detection of aa66–75 peptide of only type 1 VP13 by the Mab. The peptides were used to coat the plate at a final concentration of 10 μg·mL^−1^; ***: *p* < 0.001; (**B**) The two HEV-positive human serum samples HS392 and HS393 react with type 1 and type 4 VP13 peptide, respectively. The serum samples were diluted at 1:10 for this assay.

Since the linear and surface-oriented epitope locates in peptide aa66–75, we reasoned that it may be able to differentiate between infections by different HEV genotypes. To test this, we first had the peptides aa66–75 of all four genotypes (Figure 4A) synthesized and tested them in ELISA with the Mab. The monoclonal anti-VP13 clone 3C3 reacted only with the coating peptides of type 1 VP13 with a significant higher signal to noise (S/N) ratio than other types (Figure 6A).

Two HEV-positive serum samples were also tested using the peptide ELISA. The samples were from patients with acute HEV infection and confirmed to have HEV antibodies with a commercial ELISA kit that detects HEV antibodies against the capsid protein. The peptide ELISA showed that serum sample HS392 reacted with the type 1 VP13 peptide 66–75, and HS393 reacted with the type 4 VP13 peptide (Figure 6B). The results suggest that the genotype-specific peptide can differentiate between antibodies against the different HEV types, which may assist genotype identification in diagnosis of HEV infection.

## 4. Discussion

In this study, we identified a linear and surface-oriented epitope of VP13 of HEV. The linear epitope locates on residues aa66–75 of VP13 of genotype 1 HEV. This peptide aa66–75 can differentiate between antibodies against different HEV genotypes.

The detection of MBP-VP13 in WB by the Mab, but not MBP-X or MBP-helicase proteins, indicates the specificity of reaction. The VP13 expressed in HEK293 cells was also detected in WB with the Mab, which suggests that the epitope on VP13 is linear as the SDS-PAGE was run under denatured condition. The Mab detection of VP13 in IFA suggests that the linear epitope of VP13 is oriented on the surface of the protein. The surface orientation of the linear epitope on VP13 of genotype 1 HEV is interesting. Domain mapping and epitope screening showed that the epitope is located in residues aa66–75. The whole 10 aa appear to be required, as the truncated VP13 containing either aa66–70 or aa70–75 were not detected by the Mab.

The residue M70 appears to be essential for the epitope, as mutant M70I of type 1 peptide failed to react with the Mab. Adjacent residues may be also important as mutant T70M of type 2 and I70M of type 3 did not react with the Mab, though the residue 70 was mutated to methionine. The mutant I70M of type 3 has seven identical residues with type 1, which suggests that the other three residues also contribute to the epitope interaction with the Mab. Sequence analysis showed that aa66–75 of VP13 of genotype 1 HEV is a proline-rich region, which has a PXXP motif, but not in VP13 of other three genotypes. Our results suggest that the PXXP motif and the epitope overlap, as VP13 of the other three genotypes neither have the motif nor react with the Mab.

SH3 domains are known to bind to proline-rich sequences containing a core PXXP motif flanked by a positively charged residue [19,20]. SH3 domains comprise of about 60 residues and proteins containing SH3 domains typically play a role in signaling pathways involved in cell growth, differentiation and other regulatory functions [27]. The consensus motif recognized by class I SH3 domains is +XXPXXP (+ is either arginine or lysine, X can be any aa), and the motif for class II SH3 domains is PXXPX+ in the opposite orientation [20,27]. There are two proline-rich regions in VP13 of HEV Sar55, a genotype 1 strain: the first one locates in aa66–75 and the second one spans aa95–102. The first proline-rich region contains residues PMSPLR, a typical motif (PXXPX+) for class II SH3 domains. This PXXP motif in the first proline-rich region only exists in the VP13 of genotype 1 HEV, not the other three genotypes. The residues in the second proline-rich region are RPSAPPLP, containing an additional residue than the typical motif (+XXPXXP) for class I SH3 domains. But interestingly only the PXXP motif in the second proline-rich region is known to interact with SH3 domains [28]. The PSAP motif in the second proline-rich region is highly conserved across the four HEV genotypes that infect humans and is known to be necessary for virion release [16]. A PSAP motif in aa67–70 of avian HEV VP13 is also required for virion release [17]. The function of the PXXP motif in the first proline-rich region (aa66–75) of VP13 of genotype 1 HEV is unknown. It might play a role in cellular signaling as proline-rich motifs are also involved in interacting with other domains besides SH3 [27].

The linear epitope of VP13 was detected in HEV-infected liver cells. This is consistent with IFA detection in HEK293 cells transiently transfected with VP13 plasmid. It also confirms the surface orientation of the epitope on VP13 in the HEV-infected cells. The peptide aa66–75 of VP13 is genotype-specific, as shown by interaction with the monoclonal anti-VP13 clone 3C3. Using the peptides in ELISA can assist the identification of HEV genotypes in HEV infection. In this study, two HEV-positive serum samples were tested in the peptide ELISA. It is interesting to find out that one serum sample is from a patient infected with type 1 HEV, and the other is from a type 4 HEV-infected patient. Further study with more human serum samples is needed to verify the specificity and sensitivity of the assay.

## 5. Conclusions

A linear and surface-oriented epitope is identified on VP13 of genotype 1 HEV using a homemade monoclonal antibody. This linear epitope is located in a proline-rich region and overlaps with a PXXP motif in peptide aa66–75. The peptide aa66–75 can differentiate between antibodies against different HEV genotypes. This genotype-specific epitope should be useful in studying the function of VP13 and HEV pathogenesis.

## Figures and Tables

**Table 1:** List of primers used in this study.

Primer ^a^	Sequences (5′ to 3′) ^b^	Used for
**VP13-F1**	G*CTCGAG*GTTCGCGACCATGCGCCCTC	Cloning of VP13-D5
**VP13-R1**	C*GAATTC*TTACATCGGGGGCGAAGGGGTTG	Cloning of VP13-D5
**VP13-R2**	C*GAATTC*TTACGGCCGCAGCGGTGACATCG	Cloning of VP13-D6
**VP13-R3**	C*GAATTC*TTACACGAGGTCCAGCCCCGGCC	Cloning of VP13-D7
**VP13-R4**	C*GAATTC*TTAGGGCGGGTTGGCGAACACG	Cloning of VP13-D8
**VP13-R5**	C*GAATTC*TTACGGAGCCGAGTGGTCGGGCG	Cloning of VP13-D9
**VP13-F3**	G*CTCGAG*GTATGTCACCGCTGCGGCCGG	Cloning of VP13-D10
**VP13-R6**	C*GAATTC*TTAGCGGCGCGGCCCCAGC	Cloning of VP13-D10
**VP13-F2**	G*CTCGAG*GTATGCCACCGCTGCGGCCGG	Cloning of VP13-D10 mutant
**H3F30**	*AATTC*CCTTCGCCCCCGATGTCACCGCTGCGGCCGC	Cloning aa66–75
**H3R27**	*TCGAG*CGGCCGCAGCGGTGACATCGGGGGCGAAGGG	Cloning aa66–75
**H3F31**	*AATTC*CCTTCGCCCCCGATCTCACCGCTGCGGCCGC	Cloning aa66–75 with mutation M70I
**H3R28**	*TCGAG*CGGCCGCAGCGGTGAGATCGGGGGCGAAGGG	Cloning aa66–75 with mutation M70I
**T2H3F3**	*AATTC*CCTTTGCCCCAGATGTTGCCGCTGCGTCCGC	Cloning aa66–75 of type 2 VP13 with mutation T70M
**T2H3R3**	*TCGAG*CGGACGCAGCGGCAACATCTGGGGCAAAGGG	Cloning aa66–75 of type 2 VP13 with mutation T70M
**KH3F5**	*AATTC*CCTTCGCCGCCGATGTCGTTTCACAATCCGC	Cloning aa66–75 of type 3 VP13 with mutation I70M
**KH3R6**	*TCGAG*CGGATTGTGAAACGACATCGGCGGCGAAGGG	Cloning aa66–75 of type 3 VP13 with mutation I70M

^a^ F: forward primer; R: reverse primer. The “H3” before a primer name indicates the primer is based on sequences of HEV open reading frame 3 (ORF3) of Sar55 strain (GenBank accession number AF444002); “T2H3” indicates primer of type 2 HEV VP13 from Mexican strain (GenBank accession number M74506); KH3R6 indicates primer of type 3 HEV VP13 from Kernow-C1 strain (GenBank accession number HQ709170); ^b^ The italicized letters indicate restriction enzyme cleavage sites for cloning.
